# Deletion of the *hfsB* gene increases ethanol production in *Thermoanaerobacterium saccharolyticum* and several other thermophilic anaerobic bacteria

**DOI:** 10.1186/s13068-017-0968-9

**Published:** 2017-11-30

**Authors:** Ayşenur Eminoğlu, Sean Jean-Loup Murphy, Marybeth Maloney, Anthony Lanahan, Richard J. Giannone, Robert L. Hettich, Shital A. Tripathi, Ali Osman Beldüz, Lee R. Lynd, Daniel G. Olson

**Affiliations:** 1 Department of Biology, Molecular Biology Research Laboratories, Faculty of Art and Science, Recep Tayyip Erdogan University, Rize, Turkey; 2 Thayer School of Engineering, Dartmouth College, 14 Engineering Drive, Hanover, NH 03755 USA; 3 Biosciences Division, Oak Ridge National Laboratory, Oak Ridge, TN USA; 4 BioEnergy Science Center, Oak Ridge National Laboratory, Oak Ridge, TN USA; 5 Mascoma Corporation, Lebanon, NH USA; 6 Department of Biology, Faculty of Science, Karadeniz Technical University, Trabzon, Turkey; 7 Department of Biological Sciences, Dartmouth College, Hanover, NH USA

**Keywords:** *Thermoanaerobacterium saccharolyticum*, *Clostridium thermocellum*, *Thermoanaerobacter mathranii*, *Thermoanaerobacterium thermosaccharolyticum*, *Thermoanaerobacterium xylanolyticum*, Hydrogenase, Ethanol

## Abstract

**Background:**

With the discovery of interspecies hydrogen transfer in the late 1960s (Bryant et al. in Arch Microbiol 59:20–31, 1967), it was shown that reducing the partial pressure of hydrogen could cause mixed acid fermenting organisms to produce acetate at the expense of ethanol. Hydrogen and ethanol are both more reduced than glucose. Thus there is a tradeoff between production of these compounds imposed by electron balancing requirements; however, the mechanism is not fully known.

**Results:**

Deletion of the *hfsA* or *B* subunits resulted in a roughly 1.8-fold increase in ethanol yield. The increase in ethanol production appears to be associated with an increase in alcohol dehydrogenase activity, which appears to be due, at least in part, to increased expression of the *adhE* gene, and may suggest a regulatory linkage between *hfsB* and *adhE*. We studied this system most intensively in the organism *Thermoanaerobacterium saccharolyticum*; however, deletion of *hfsB* also increases ethanol production in other thermophilic bacteria suggesting that this could be used as a general technique for engineering thermophilic bacteria for improved ethanol production in organisms with *hfs*-type hydrogenases.

**Conclusion:**

Since its discovery by Shaw et al. (JAMA 191:6457–64, 2009), the *hfs* hydrogenase has been suspected to act as a regulator due to the presence of a PAS domain. We provide additional support for the presence of a regulatory phenomenon. In addition, we find a practical application for this scientific insight, namely increasing ethanol yield in strains that are of interest for ethanol production from cellulose or hemicellulose. In two of these organisms (*T. xylanolyticum* and *T. thermosaccharolyticum*), the ethanol yields are the highest reported to date.

**Electronic supplementary material:**

The online version of this article (10.1186/s13068-017-0968-9) contains supplementary material, which is available to authorized users.

## Background

Thermophilic bacteria have long been studied for their potential use in biofuel production from cellulose and hemicellulose. Microbes that have received particular study in this context include the cellulose-fermenting *Clostridium thermocellum* as well as hemicellulose-fermenting organisms including *Thermoanaerobacterium saccharolyticum, Thermoanaerobacterium thermosaccharolyticum*, *Thermoanaerobacterium xylanolyticum*, and *Thermoanaerobacter mathranii.* These, and many other obligate and facultative anaerobic microbes carry out a mixed acid fermentation, whereby sugar is converted into a mixture of organic acids, ethanol, H_2_, and CO_2_. It is, however, stoichiometrically possible to produce two moles of ethanol per mole of C_6_ sugar (C_6_H_12_O_6_ → 2 C_2_H_6_O + 2 CO_2_) at 97% thermodynamic efficiency (based on heat of combustion, [1]). To achieve this conversion, all of the electrons initially present in the C_6_ sugar must be transferred to ethanol and not diverted to organic acids or H_2_.

The mechanism determining the distribution of products in mixed acid fermentation is in general not known. One hypothesis is that product distribution is determined by the law of mass action (i.e., that flux to different end-products is controlled by the effect of the concentration of those products on the reaction kinetics). Support for this hypothesis is found in experiments where increasing the partial pressure of H_2_ led to an increase in ethanol production [2, 3], or conversely, a decrease in ethanol production when the partial pressure of H_2_ was decreased, by introduction of a syntrophic H_2_-consuming methanogen [4, 5].

An alternative hypothesis is that product distribution is determined by a specific regulatory process. One example of such a system is Rex, a protein that modulates gene expression in response to changes in the NADH/NAD^+^ ratio [6]. It was first described in *Streptomyces coelicolor* [7], but has also been studied in members of the *Clostridium* [8] and *Thermoanaerobacter* [9] genera. Deletion of the *rex* gene has been shown to increase ethanol production [10–12].

Zheng et al. have proposed that in the mixed acid fermenter *Ruminococcus albus*, the H_2_ partial pressure is sensed by the *hydS* gene (homologous to *T. saccharolyticum hfsB*) via its hydrogenase domain and that the signal is then transduced via its PAS domain to an adjacent Ser/Thr protein kinase [13]. PAS domains are widely used in all three domains of life and frequently found as part of a signal transduction cascade involving serine/threonine protein kinase and phosphatases. In H_2_-oxidizing bacteria, such as *Ralstonia eutropha*, the mechanism of H_2_-sensing has been worked out in detail [14–16]. In the presence of H_2_, the HoxJ protein does not phosphorylate HoxA, and non-phosphorylated HoxA is a transcriptional activator of hydrogenase genes. However, the HoxABCJ hydrogenases do not share any significant homology with those of *T. saccharolyticum*. Thus, there is sequence-homology-based evidence suggesting that the *hfsB* gene in *T. saccharolyticum* could be involved in H_2_ sensing, but to date this has not been experimentally verified.

Here we seek to identify regulatory processes that control end-product formation in thermophilic bacteria. We also address the effect of mutations in *hfs* hydrogenase subunits on ethanol production in *T. saccharolyticum* as well as other thermophilic anaerobes.

## Results

**Fig. 1:**
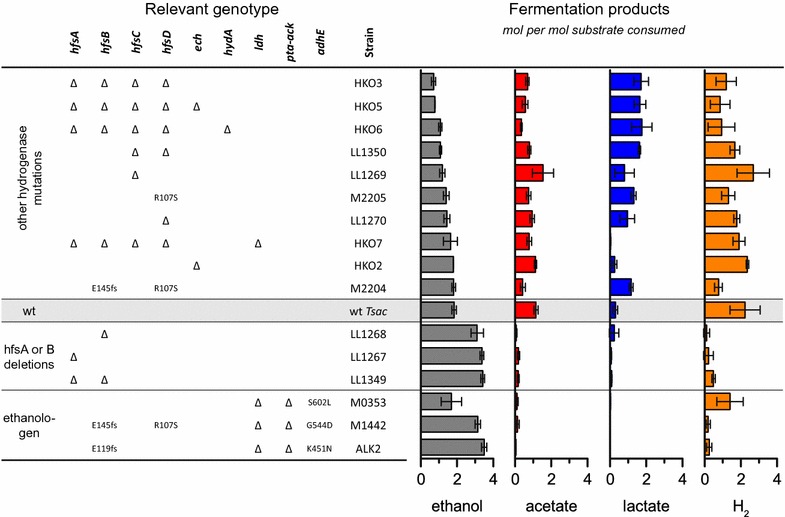
Comparison of fermentation products produced by strains of *T. saccharolyticum* with mutations in the hfs operon. Strains were grown in MTC-6 medium on 5 g/l (14.7 mM) cellobiose. Error bars represent one standard deviation, *n* ≥ 3

It has been previously observed that *T. saccharolyticum* can be engineered for increased ethanol yield by deleting pathways for lactate and acetate production [17, 18], but that ethanol production increases a few generations after the deletion of phosphotransacetylase (*pta*), acetate kinase (*ack*), and/or lactate dehydrogenase (*ldh*) rather than immediately. To try to understand this phenomenon, we resequenced the genomes of strains of *T. saccharolyticum* with deletions of *ldh* and *pta*-*ack* that exhibited both low and high ethanol yields (Fig. 1, strain M0353 vs. ALK2 and M1442 and Additional file 1: Table S1). In these strains, we observed that mutations in the *hfsB* gene were correlated with increased ethanol production. Previously, we had deleted the entire *hfsABCD* operon, and had not seen a substantial increase in ethanol production [19]. To resolve this apparent contradiction, and to better understand how the *hfs* operon works in *T. saccharolyticum*, we deleted each subunit (A, B, C, and D) one-by-one, using allelic replacement with the kanamycin antibiotic resistance marker (*kan*). (Evidence of gene deletion is presented in Additional file 2: Table S4.)

We observed that strains with deletions of the *A* and/or *B* subunits (Fig. 1, strains LL1267, LL1268, and LL1349) produced ethanol at a yield of ≥ 3 mol of ethanol per mole of cellobiose consumed, whereas strains with deletions of *C* and/or *D* subunits (Fig. 1, strains LL1269, LL1270, and LL1350) produced ethanol at a molar yield of about 2, which is equivalent to the wild-type strain (wt Tsac). Modifications to the *hfs* operon did not seem to affect growth rate or biomass formation (Additional file 3: Figure S2).

**Fig. 2:**
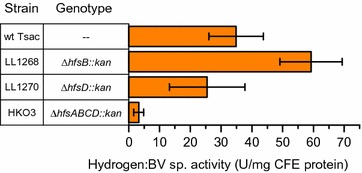
Hydrogenase activity in *T. saccharolyticum* was observed using the hydrogen: benzyl viologen enzyme assay. Error bars represent one standard deviation, *n* ≥ 3

The observation that wild-type *hfsC* and *D* subunits were necessary for high-yield ethanol production suggested a regulatory mechanism, and we performed additional experiments to determine its nature. First, we measured hydrogenase activity using a benzyl viologen assay. Although deletion of the entire *hfsABCD* operon decreased hydrogenase activity, deletion of just the *hfsB* subunit actually increased activity (Fig. 2, Additional file 4: Table S2).

**Fig. 3:**
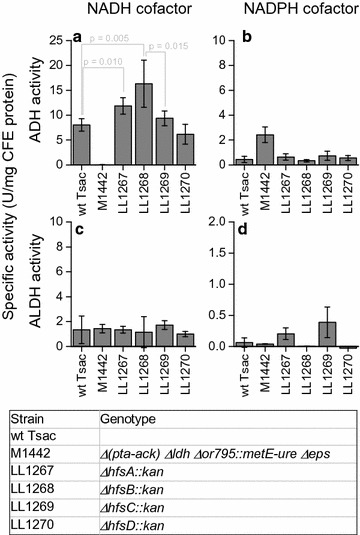
Comparison of alcohol dehydrogenase (ADH) and acetaldehyde dehydrogenase (ALDH) enzyme activities in *T. saccharolyticum* with both the NADH and NADPH cofactors. Error bars represent one standard deviation, *n* ≥ 4

Next, we looked at the enzymes involved in conversion of acetyl-CoA to ethanol. This conversion is mediated by two reactions, acetyl-CoA reduction to acetaldehyde (ALDH) and acetaldehyde reduction to ethanol (ADH). Both of these reactions can, in theory, use either NADH or NADPH as an electron donor, so we tested all four combinations (Fig. 3). We found that NADH-ADH activity was significantly increased when either *hfsA* or *hfsB* was deleted (*p* = 0.010 or 0.005 respectively).

**Fig. 4:**
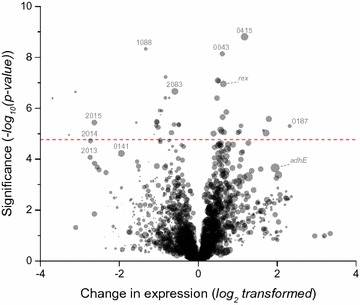
Identification of significant changes in gene expression associated with high levels of ethanol production in *T. saccharolyticum*. Gene expression was compared between the high ethanol-producing group of strains (LL1267, LL1268, and M1442) and strains that produced ethanol at wild-type levels (wt Tsac, LL1269, LL1270, and HKO3). Genes whose expression was higher in the high ethanol-producing group are plotted on the right side of the graph. The significance of the change is plotted on the vertical axis. The red dashed line indicates a *p* value of 0.05 adjusted with a Bonferroni correction for 2868 multiple tests. The size of the symbol indicates the average normalized expression value

To understand the reason for the change in enzyme activity, we looked at transcript levels for several strains including the wild-type (wt Tsac), high ethanol-producing (strain M1442), *hfsABCD* deletion (strain HKO3), and individual *hfsA*, *B*, *C*, and *D* deletions (strains LL1267, LL1268, LL1269, and LL1270) (Fig. 4). In addition to transcriptomic data, we collected some preliminary proteomic data as well. Since we observed similar patterns of expression in both datasets, we did not collect a detailed set of proteomic data. Both RNAseq and proteomic datasets are available in Additional file 5: Table S3.

**Fig. 5:**
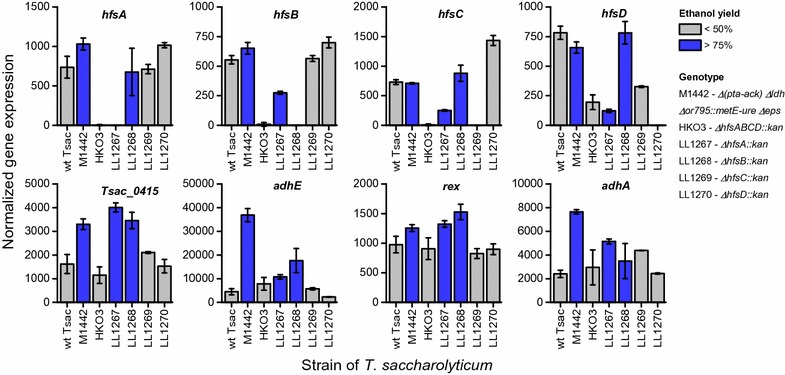
Expression levels of selected genes in several strains of *T. saccharolyticum*. Each panel shows expression data from each strain for a given gene. Expression data are from RNAseq measurements, normalized to adjust for the length of each gene and total number of reads mapped for each library. Error bars represent one standard deviation, *n* = 2–4. Bars are colored based on the ethanol yield of the strain. Blue bars indicate strains with high ethanol production (yield ≥ 75% of theoretical maximum) and gray bars indicate low ethanol production (yield ≈ 50% of theoretical maximum, similar to wild type)

Although this analysis revealed several interesting candidates, we decided to look more closely at four genes: *adhE*, *adhA*, *rex*, and *Tsac_0415* (Fig. 5). The first three were chosen because they have been previously associated with ethanol production in this organism [20, 21]. The last was chosen because it is immediately adjacent to adhE and is one of the most significant observations from Fig. 4. We also looked at expression of *hfsA*, *B*, *C*, and *D* to see if replacement of the targeted gene with the *kan* marker had any polar effects on downstream genes.

Deletion of *hfsA* reduced expression of downstream genes (*hfsB*, *C*, and *D*) by two to six-fold. Deletion of *hfsB* did not have any polar effects. Deletion of *hfsC* slightly reduced *hfsD* expression. Deletion of *hfsD* increased *hfsA* and *hfsC* expression, but did not change *hfsB* expression. Overall, the insertion of the *kan* marker did not seem to have a consistent effect on downstream genes. Expression of *Tsac_0415*, *adhE* and *rex* were all correlated with increased ethanol production (Fig. 5). The RNAseq results were confirmed by RT-qPCR for *adhE* and *adhA* (Additional file 6: Figure S3).

**Fig. 6:**
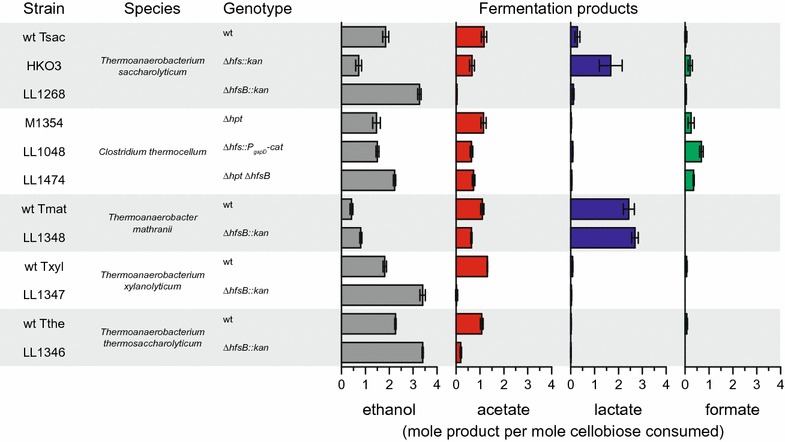
Fermentation products from various organisms with deletions of their hfs genes. Strains were grown in MTC-5 medium (*C. thermocellum*) or MTC-6 medium (other strains) on 5 g/l (14.7 mM) cellobiose. For all products, the maximum theoretical yield is 4 mol product per mole cellobiose consumed. Error bars represent one standard deviation, *n* ≥ 3

Finally, to determine if the effect of *hfsB* deletion is unique to *T. saccharolyticum*, we deleted *hfsB* in *C. thermocellum*, *T. mathranii*, *T. xylanolyticum*, and *T. thermosaccharolyticum* (Fig. 6). In all of these organisms, ethanol production increased. In *C. thermocellum*, we made a deletion of the whole *hfs* operon (Clo1313_1796–1793) in addition to the deletion of just the *hfsB* subunit. In this organism, we see a pattern similar to that which we observed for *T. saccharolyticum*: deletion of the *hfsB* subunit alone improved ethanol production, whereas deletion of the whole *hfs* operon did not.

Strains used in this work were resequenced to confirm that the genetic modification had been made as intended, to check for possible contamination and to identify the presence of secondary mutations. All strains were correct, no evidence of contamination was found. Secondary mutations in genes known to be related to fermentation product production are noted in Fig. 1. All mutations are listed in Additional file 2: Table S4 (strains of *T. saccharolyticum*) or Additional file 7: Table S5 (other strains). Raw sequencing data can be obtained based on accession numbers presented in Table 1.

**Table 1:** Strains used in this work

Name	ID	Species	Genotype	Accession number	References
M1354	LL345	*Clostridium thermocellum*	*∆hpt*	SRP053786	[34]
LL1048	LL1048		*∆hfs::PgapD*-*cat*	SRP108090	This work
LL1474	LL1474		*∆hpt ∆hfsB*	(Not sequenced)	This work
wt Tmat	LL1258	*Thermoanaerobacter mathranii*	*wt*	SRP108092	DSMZ 11426
LL1348	LL1348		*ΔhfsB::kan*	SRP108103	This work
wt Tsac	LL1025	*Thermoanaerobacterium saccharolyticum*	*wt*	SRA234880	[35]
ALK2	LL1040		*∆ldh::erm ∆(pta*-*ack)::kan*	SRP084787	[18]
M1442	LL1049		*∆(pta*-*ack) ∆ldh Δor795::metE*-*ure Δeps*	SRP052455	[36]
M0353	LL1143		*∆pyrF ∆(pta*-*ack) ∆ldh*	SRP084629	[17]
HKO2	LL1186		*Δech::erm*	SRP085666	[19]
HKO3	LL1187		*Δhfs::kan*	SRP085668	[19]
HKO5	LL1188		*Δech::erm Δhfs::kan*	SRP086313	[19]
M2204	LL1189		hfs::hfs*-*kan*	SRP085677	[19]
HKO7	LL1190		*Δhfs::kan ∆ldh::erm*	SRP085674	[19]
HKO6	LL1191		*Δhfs::kan ΔhydA::erm*	SRP085673	[19]
M2205	LL1195		*hfs::hfsDR107S*-*kan*	SRP086210	[37]
LL1267	LL1267		*ΔhfsA::kan*	SRP108081	This work
LL1268	LL1268		*ΔhfsB::kan*	SRP108082	This work
LL1269	LL1269		*ΔhfsC::kan*	SRP108085	This work
LL1270	LL1270		*ΔhfsD::kan*	SRP108087	This work
LL1349	LL1349		*ΔhfsAB::kan*	SRP108105	This work
LL1350	LL1350		*ΔhfsCD::kan*	SRP108109	This work
wt Tthe	LL1244	*Thermoanaerobacterium thermosaccharolyticum*	*wt*	SRP096459	DSMZ 571
LL1346	LL1346		*ΔhfsB::kan*	SRP108099	This work
wt Txyl	LL1301	*Thermoanaerobacterium xylanolyticum*	*wt*	SRP108095	DSMZ 7097
LL1347	LL1347		*ΔhfsB::kan*	SRP108102	This work

## Discussion

In this work, we set out to answer three questions:

1. How is the distribution of fermentation products controlled in *T. saccharolyticum*?
2. What is the effect of mutations in the *hfsABCD* operon in *T. saccharolyticum*?
3. Is modification of the *hfs* operon a technique that can be used to increase ethanol production in other organisms?

We believe our data add support to the hypothesis that a regulatory phenomenon of some type controls the distribution of fermentation products in *T. saccharolyticum* (i.e., flux distribution is not solely determined by mass action), since deletion of the *hfsA*, *B, or AB* subunits reduces H_2_ production to a greater extent than a complete deletion of the *A*, *B*, *C,* and *D* subunits. If no regulatory phenomenon was present, we would expect a complete deletion (*ABCD*) to have a similar or more extreme phenotype (i.e., greater reduction in H_2_ production) to the *A* and *B* deletions, but we observe the opposite result.

To better understand the regulatory cascade, we need to consider the other fermentation products. Deletion of the *hfsA* and *B* subunits diverts flux toward ethanol production, whereas the deletion of the *hfsC* or *D* subunits diverts flux toward lactate production. Lactate is often considered to be a product of “overflow” metabolism. In *T. saccharolyticum* (and many other organisms), this enzyme is allosterically regulated by fructose 1,6 bisphosphate (FBP) [22]. When cells are growing rapidly, FBP levels are low and the enzyme is inactive. When FBP accumulates, the enzyme is activated and lactate is produced.

In *T. saccharolyticum*, ethanol production is almost exclusively mediated by two enzymes, *AdhE* and *AdhA* [20], thus one or both of these enzymes are likely the final target of the regulatory cascade. Based on transcription data, we do not observe a correlation between changes in *adhA* expression and deletion of *hfs* subunits (Fig. 6). Furthermore, the AdhA enzyme responsible for NADPH-linked ADH activity and changes in this activity are also uncorrelated with deletion of *hfs* subunits (Fig. 4). These two lines of evidence suggest that *adhA* does not participate in regulation of ethanol production by *hfs*. This leaves *adhE* as a possible target of *hfs* (either directly or indirectly). There is some indication that regulation of *adhE* takes place at the transcript level. Strains with deletions of the *hfsA* or *B* subunits show a two- and threefold increase in *adhE* transcript levels, whereas strains with deletions of the *hfsC* or *D* subunits show no significant change in *adhE* expression (Fig. 6). The changes in *adhE* expression, however, cannot explain all of the observed changes in enzyme activity. AdhE is a bi-functional enzyme, with both ADH and ALDH enzyme activities [21]. In the wild-type strain, both activities are linked to the NADH cofactor. If changes in *adhE* expression were the sole cause of changes in ethanol production, we would expect to see NADH-ADH and NADH-ALDH activity increase by similar amounts. Instead we observe a significant increase in NADH-ADH activity (*p* = 0.005), but no change in NADH-ALDH activity. This suggests that regulation may also take place at the post-transcriptional level.

There are a few possibilities for other components which might mediate signal transduction between *hfsCD* and *adhE*. One of these is *rex*, a gene that has been shown to regulate *adhE* expression in response to changes in the NADH/NAD^+^ ratio in many organisms [9]. The Rex protein typically functions by binding to DNA upstream of a target gene and inhibiting its expression. The presence of an excess of NADH disrupts the Rex-DNA binding and allows transcription. Thus, deletion of *rex* should result in increased ethanol production, and overexpression of *rex* (assuming NADH/NAD^+^ ratio is unchanged) should result in a decrease in ethanol production. We see the opposite pattern in our expression data: strains with increased ethanol production show increased levels of *rex* expression (Figs. 5 and 6).

Another component that may play a role in the regulatory cascade is the *tsac_0415* gene. This gene is notable because it shows the most significant difference in expression between high and low ethanol-producing strains (Fig. 5). It is also directly adjacent to *adhE* on the chromosome (*tsac_0416* locus), although they are transcribed in opposite directions. This protein is annotated by PFAM as “unknown function [23].” A BLAST search revealed only 5 close matches in the nr database, all in the *Thermoanaerobacterium* genus [24]. Interestingly, this set of matches includes both *T. xylanolyticum* and *T. thermosaccharolyticum* (strains that show a large increase in ethanol production when *hfsB* is deleted), but not *C. thermocellum* or *T. mathranii* (strains which show only a moderate increase in ethanol production when *hfsB* is deleted). Confirmation of this hypothesis awaits further experimental evidence.

In the course of trying to understand the effect of mutations in the *hfs* operon, we discovered that our strains exhibited different distributions of fermentation products compared to what has been previously reported [19]. For example, Shaw et al. reported that a deletion of *hfsABCD* resulted in a 96% decrease in H_2_ production, whereas we observed only a 53% decrease (Fig. 1). Since, for this comparison, we used the strain from Shaw et al. we suspect the most likely reason for the difference is a difference in fermentation conditions. Shaw et al. used rich media (DSM 122) whereas we used a chemically defined medium (MTC-6). Another possibility is the accumulation of secondary mutations; however, our resequencing data of that strain shows only five mutations in addition to the targeted *hfsABCD* deletion (Additional file 2: Table S4, and none of those mutations seem to affect genes related to fermentation). A final possibility we considered was that our strains were contaminated with wild-type *T. saccharolyticum*. We excluded this possibility by PCR of both internal and external regions of the *hfs* locus (Additional file 8: Figure S1), and whole genome resequencing (Additional file 2: Table S4).

Although we have shown that deletions of *hfsA* and/or *B* in *T. saccharolyticum* are sufficient to generate a high-yielding ethanologen strain, we wanted to see if this phenomenon could be generalized to other organisms. We found that *hfsB* deletions resulted in large increases in ethanol production in *T. xylanolyticum* and *T. thermosaccharolyticum*, but there was at least some increase in ethanol production in all strains tested (Fig. 6). Thus, we have shown that the deletion of *hfsB* is a technique which can be used to increase ethanol production in a variety of organisms that are of interest for biofuel production.

## Methods

### Identification of candidate organisms for *hfsB* deletion

We applied the following criteria to select candidate organisms for testing the universality of whether the *hfsB* deletion increases ethanol production.

1. The presence of all four *hfs* subunits (A, B, C, and D).
2. The presence of *nfnAB* and *adhA* genes.
3. Known transformation systems.

*C. thermocellum* does not strictly adhere to these criteria (its *hfs* operon structure is slightly different and it does not have an *adhA* gene), but it is an organism that is of interest for biofuel production, has a well-defined genetic system, and is much different than the *Thermoanaerobacter* and *Thermoanaerobacterium* species identified above. Also *T. mathranii* does not have an *hfsA* subunit, and the orientation of other genes in the operon is different.

### Strains and chemicals

All reagents used in this study were of molecular grade, and obtained either from Sigma Aldrich or Fisher Scientific, unless otherwise noted. For all fermentations, cellobiose was used as the primary carbon source at a concentration of 5 g/l, unless otherwise noted. *C. thermocellum* strains were grown at 55 °C under anaerobic conditions, either in conical tubes in anaerobic chambers (Coy Laboratory Products, Grass Lakes, MI, USA). *C. thermocellum* DSM1313 was obtained from the DSMZ culture collection. It was cultured in either MTC-5 (chemically defined) medium at a pH of 7.4 [25] or CTFUD (rich) medium [26] at a pH of 7.0. *T. saccharolyticum, T. xylanolyticum, T. thermosaccharolyticum, and T. mathranii* were grown on MTC-6 (chemically defined) medium [27] or CTFUD medium. For *T. saccharolyticum* and *T. xylanolyticum*, the pH was 6.2 for routine culture. For *T. thermosaccharolyticum*, the pH was 7.0, while for *T. mathranii* it was 7.4.

For transformation, *T. saccharolyticum*, *T. xylanolyticum*, *T. thermosaccharolyticum*, and *T. mathranii* strains were grown anaerobically at 55 °C, with an initial pH of 6.2 (for *T. saccharolyticum* and for *T. xylanolyticum*) and 6.7 (for *T. thermosaccharolyticum* and for *T. mathranii*). Transformations were performed in CTFUD media (pH 6.7) which contained, 10 g/l xylose instead of cellobiose.

### Molecular biology and strain construction

Transformation of *T. saccharolyticum*, *T. mathranii*, *T. xylanolyticum*, and *T. thermosaccharolyticum* was performed in an anaerobic chamber using the natural competence method [28]. Transformation of *C. thermocellum* was performed using electroporation as described previously [26]. *T. saccharolyticum* was selected on 200 µg/ml kanamycin, while *T. xylanolyticum* and *T. thermosaccharolyticum* were selected on 500 µg/ml kanamycin *and T. mathranii* was selected on 1000 µg/ml kanamycin*. C. thermocellum* was selected on 6 µg/ml thiamphenicol.

### Genetic modifications in *T. saccharolyticum*, *T. thermosaccharolyticum*, *T. xylanolyticum,* and *T. mathranii*

The GenBank accession number for the complete nucleotide sequence of the *hfs* operon is GQ354412. Amino acid sequences of *hfsA*, *hfsB*, *hfsC*, and *hfsD* were also retrieved from NCBI with the accession numbers ACU11594.1, ACU11595.1, ACU11596.1, and ACU11597.1 respectively. Genetic modification of these organisms was performed using linear DNA fragments constructed via isothermal DNA assembly (Gibson Assembly) [29, 30]. In general, each construct consisted of a 3′ homology flank, selection marker, and 5′ homology flank. Primer sequences are presented in Additional file 9: Table S6. Assembly fragments were generated by PCR with Phusion HiFi Master Mix (New England Biolabs) in 50 µl reaction volume containing 2 µl cell culture, 1X Phusion HiFi Master Mix, 0.5 µM each primer, with the following conditions: initial denaturation steps at 98 °C for 5 min, denaturation at 98 °C for 10 s, followed by annealing at 54–60 °C (depending on the primer’s Tm) for 30 s and primer extension at 72 °C for 40 s—1 min (depending on the fragment size), followed by a step at 72 °C for 10 min, for 34 cycles. The resulting fragments were assembled using a 1:1:1 ratio in 20 µl NEB HiFi DNA Assembly Master Mix (New England Biolabs), at 55 °C for 20 min. PCR evidence for successful strain construction is presented in Additional file 8: Figure S1.

### Genetic modification of *C. thermocellum*

In this study, a genetically tractable Δ*hpt* strain of *C. thermocellum* DSM1313 (referred as LL345) was used for subsequent genetic modifications [22]. Genetic loci of the *hpt* and *hfsB* genes are Clo1313_2927 and Clo1313_1795, respectively. The deletion plasmid was designed to contain three ~ 1000 bp regions homologous to the upstream (5′ flank), downstream (3′ flank), and internal regions of the *hfsB* gene on the *C. thermocellum* chromosome. The vector backbone was amplified by PCR using the pDGO145 plasmid as the template (GenBank accession number: KY852359). The homology fragments were amplified by PCR with Phusion polymerase. Fragments were assembled via Gibson assembly and cloned in *E. coli* NEB5α cells. The resulting plasmid (pLL1192) was purified using the QIAprep Spin Miniprep Kit (Qiagen) in accordance with the manufacturer’s directions and transformed into the *E. coli* T7 express cells to ensure proper methylation [31]. The plasmid was then transformed into *C. thermocellum* strain LL345 via electroporation as described previously [26].

### Fermentation conditions

#### Analytical chemistry

Cellobiose, glucose, pyruvate, formate, acetate, ethanol, succinate, and lactate were measured by HPLC using an Aminex HPX-87H column (BioRad, CA, USA) with refractive index and ultraviolet detectors as described previously [27]. Hydrogen gas was measured by gas chromatograph using a thermal conductivity detector and nitrogen as the carrier gas, as described previously [27].

### Enzyme assays

#### Cell-free extract (CFE) preparation

Cells were grown in CTFUD medium to mid-log phase (OD_600_ = 0.3–0.8). Note: we initially tried growing cells in MTC-6 medium for enzyme assays, but had problems with incomplete cell lysis. We did not have problems with cell lysis when cells were grown in CTFUD medium and thus all enzyme assay data reported here are from cells grown in CTFUD medium. After growth, cells were separated by centrifugation at 6000×*g* for 15 min at room temperature (~ 25 °C). The supernatant was discarded and the cell pellet was washed two times with 1 ml of a buffer containing 100 mM Tris–HCl (pH 7.5 at 25 °C) and 2 mM dithiothreitol (DTT). The cell pellet was resuspended in a final volume of 200–1000 µl of the wash buffer. A volume of 3 µl (4000–8000 U) of Ready-Lyse lysozyme enzyme (Epicenter) was added and cells were incubated at room temperature for 30 min or until an increase in viscosity (due to DNA) was observed. Then 2 µl DNAse I (Thermo Fisher) was added to reduce the viscosity of the solution and it was incubated for an additional 20 min. The resulting solution was centrifuged at 10,000×*g* for 5 min at room temperature and the supernatant was used as cell-free extract (CFE) for enzyme assays. CFE was used immediately or stored at 4 °C for up to 1 week.

The initial centrifugation step was performed aerobically, although the tubes were kept tightly capped when outside of the anaerobic chamber. The subsequent washes and all other steps were performed in a COY anaerobic chamber (COY labs, Grass Lake, MI). The anaerobic atmosphere was 10% CO_2_, 1–3% hydrogen, and the balance nitrogen. Anaerobic conditions were maintained (less than 5 ppm oxygen) with a palladium catalyst.

### General assay conditions

Enzyme assays were performed as described previously [27]. Briefly, enzyme activity was assayed in an anaerobic chamber (COY labs, Grass Lake, MI) using an Agilent 8453 spectrophotometer with an external water bath to maintain assay temperature. The units for all enzyme activities are expressed as μmol of product min^−1^ (mg of cell extract protein)^−1^. For each enzyme assay, at least two concentrations of cell extract were used to confirm that specific activity was proportional to the amount of extract added. All chemicals and coupling enzymes were purchased from Sigma except for coenzyme A, which was purchased from EMD Millipore (Billerica, MA). All chemical solutions were prepared fresh weekly.

### Alcohol dehydrogenase (ADH) and aldehyde dehydrogenase (ALDH) assays

The reaction was performed in 1.2 ml total volume in reduced-volume quartz cuvettes (part number 29MES10; Precision Cells Inc., NY) with a 1.0 cm path length. For ADH (acetaldehyde reduction) and ALDH (acetyl-CoA reduction) reactions, the anaerobic reaction mixture contained 50 mM Tris–HCl buffer (pH 7.5), 0.3 mM NADPH or NADH, 10 mM acetaldehyde (ADH) or 1 mM acetyl-CoA (ALDH), 2 mM MgCl_2_, and 1 mM DTT. Specific activities were evaluated by decrease in absorbance at 340 nm caused by NADPH or NADH oxidation at 55 °C inside an anaerobic chamber. Reactions were monitored by an Agilent 8453 UV–vis spectrophotometer (with temperature controlled by a water bath outside of the anaerobic chamber). Reaction was initiated with the addition of acetaldehyde or acetyl-CoA. The background activity was determined by measuring the slope of the change in absorbance reaction before the reaction was initiated.

### Hydrogenase assay

Hydrogenase assays were performed anaerobically at 60 °C in anaerobic microcuvettes with rubber stoppers (Starna Cells, Atascadero, CA) sealed in an anaerobic chamber (COY Labs, Grass Lake, MI) with an atmosphere of ~ 89% N_2_, 10% CO_2_, and 1% H_2_. Initial rates of benzyl viologen (BV) reduction were recorded with a spectrophotometer at 578 nm (ε = 8.65 mM^−1^ cm^−1^) [19, 32]. The reaction was set up in a 1 ml final volume containing 50 mM Tris–HCl (pH 7.5), 1.0 mM BV, and 0.2–0.06 µg of protein and was initiated with the addition of 0.02 mmol of hydrogen to the cuvette headspace [19].

### Transcript analysis

#### RNA preparation

RNA was prepared from 10 ml mid-log-phase cells grown in MTC-6 medium. Mid-log phase was determined individually for each culture. For wild-type *T. saccharolyticum* and HKO3, the OD_600_ at harvest was 0.6–0.8. For LL1267, LL1269, and LL1270, it was 0.1–0.2. For LL1268 and M1442, it was 0.3–0.4. At harvest, the culture was treated with 20 ml RNA Protect Bacteria Reagent (Qiagen). After centrifugation, pellets were stored at − 80 °C until RNA purification. RNA was extracted by RNeasy mini kit (Qiagen) and contaminated DNA was removed using RNase-Free DNase set (Qiagen). The resulting RNA was quantified using a Qubit 2.0 fluorometer using Life Technologies Quant-iT™ RNA Assay Kit. The standard curve contained three points (50, 400, and 1000 ng/µl) and a blank. The quality was analyzed using the Agilent 2100 Bioanalyzer. RNA purity was determined with NanoDrop spectrophotometer, by measuring the ratio of absorbance at 260 nm vs 280 nm.

#### RNAseq analysis

For RNAseq analysis, RNA (prepared as described above in the “RNA preparation” section) was sent to the Joint Genome Institute (Walnut Creek, CA) for conversion to cDNA and Illumina sequencing. The Illumina sequencing was performed as described in the “Illumina sequencing” section in the Additional file 10: Additional methods.

Data were normalized as follows: raw FASTQ file reads were filtered and trimmed using the JGI QC pipeline resulting in the filtered FASTQ file (*.anqrptk.fastq.gz files). Using BBDuk (http://jgi.doe.gov/data-and-tools/bbtools/bb-tools-user-guide/bbduk-guide/), raw reads were evaluated for artifact sequence by kmer matching (kmer = 25), allowing 1 mismatch and detected artifact was trimmed from the 3′ end of the reads. RNA spike-in reads, PhiX reads, and reads containing any Ns were removed. Quality trimming was performed using the phred trimming method set at Q10. Following trimming, reads under the minimum length threshold of 45 bases were removed.

To identify genes associated with increased ethanol production, we compared the high ethanol-yielding strains (M1442, LL1267, and LL1268) with low ethanol-yielding strains (wt, HKO3, LL1269, and LL1270) using a two-tailed *t* test. Data are presented as a volcano plot (Fig. 4), a type of scatter-plot used to quickly identify changes in large datasets composed of replicate data [33]. The vertical axis represents the significance (log-transformed *p* value, with the most significant values near the top of the plot). The horizontal axis represents fold-change (log-transformed so that increases and decreases in expression are given equal visual weight). Raw data used to generate the figure are presented in Additional file 5: Table S3.

## Additional files

**Additional file 1: Table S1.** Fermentation data.

**Additional file 2: Table S4.** *T. saccharolyticum* resequencing data.

**Additional file 3: Figure S2.** Growth curves.

**Additional file 4: Table S2.** Enzyme assay data.

**Additional file 5: Table S3.** RNAseq and protomic data.

**Additional file 6: Figure S3.** Expression of *adhE* and *adhA* genes determined by RT-qPCR.

**Additional file 7: Table S5.** Resequencing data from other strains.

**Additional file 8: Figure S1.** Confirmation of hydrogenase deletions in HKO strains.

**Additional file 9: Table S6.** List of primers.

**Additional file 10: Additional methods.** Additional methods.
