# Supplementary material for: Deletion of the hfsB gene increases ethanol production in Thermoanaerobacterium saccharolyticum and several other thermophilic anaerobic bacteria
Source: Biotechnol Biofuels. 2017 Nov 30;10:282. doi: 10.1186/s13068-017-0968-9 (PMC5707799; doi:10.1186/s13068-017-0968-9)
Supplement: Supplementary file 3 — Additional file 3: Figure S2. Growth curves. [file 13068_2017_968_MOESM3_ESM.pdf]

Figure S2. Growth curves

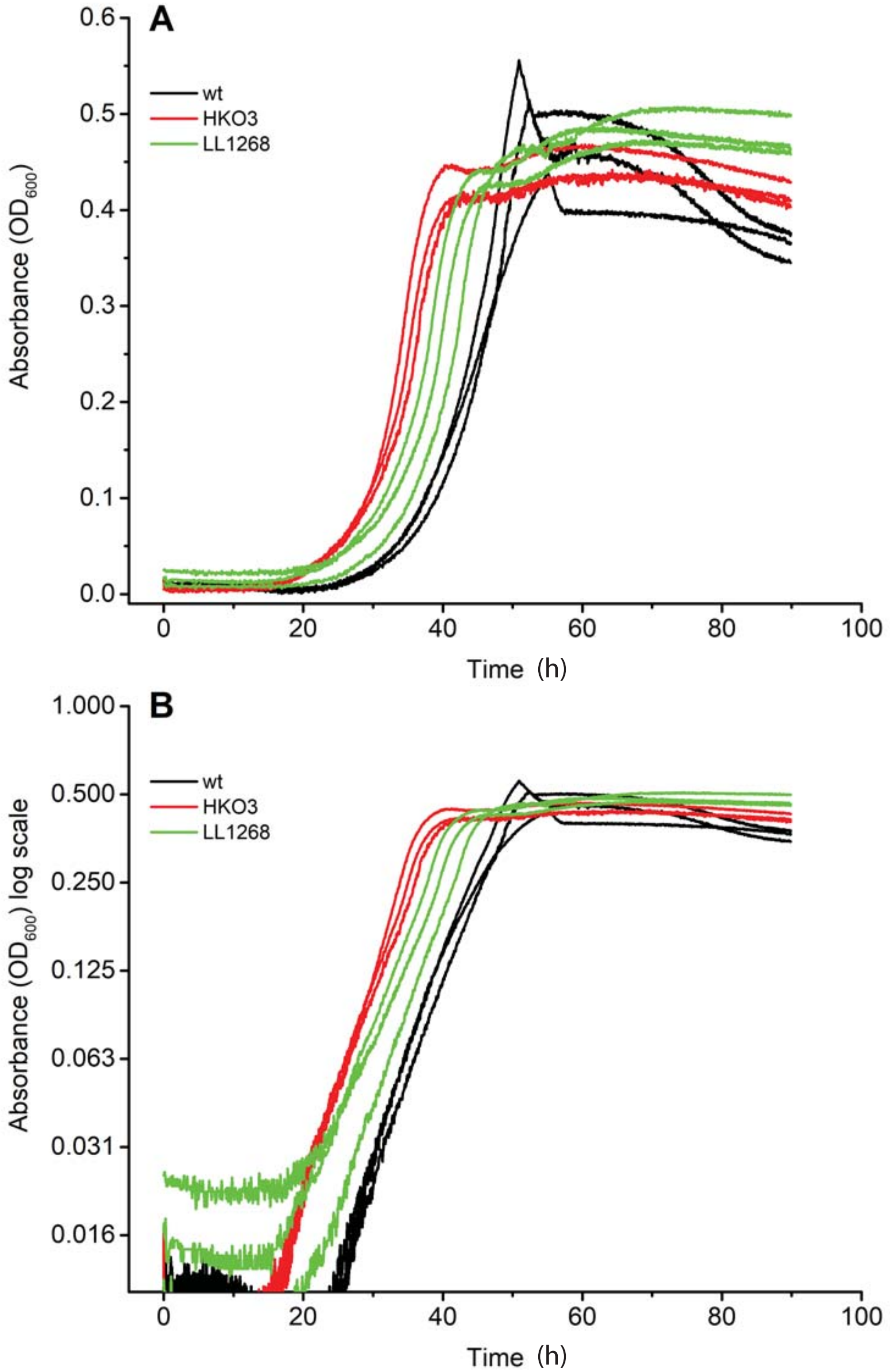

Growth curves are shown for the wild type, HKO3 (*hfsABCD* deletion) and LL1268 (*hfsB* deletion) strains). Curves are shown with either a linear scale (panel **A**) or a log2 scale (panel **B**) for comparision of maximum growth as well as growth rates.
