# Supplementary material for: Deletion of the hfsB gene increases ethanol production in Thermoanaerobacterium saccharolyticum and several other thermophilic anaerobic bacteria
Source: Biotechnol Biofuels. 2017 Nov 30;10:282. doi: 10.1186/s13068-017-0968-9 (PMC5707799; doi:10.1186/s13068-017-0968-9)
Supplement: Supplementary file 6 — Additional file 6: Figure S3. Expression of adhE and adhA genes determined by RT-qPCR. [file 13068_2017_968_MOESM6_ESM.pdf]

Figure S3. Expression of *adhE* and *adhA* genes determined by RT-qPCR

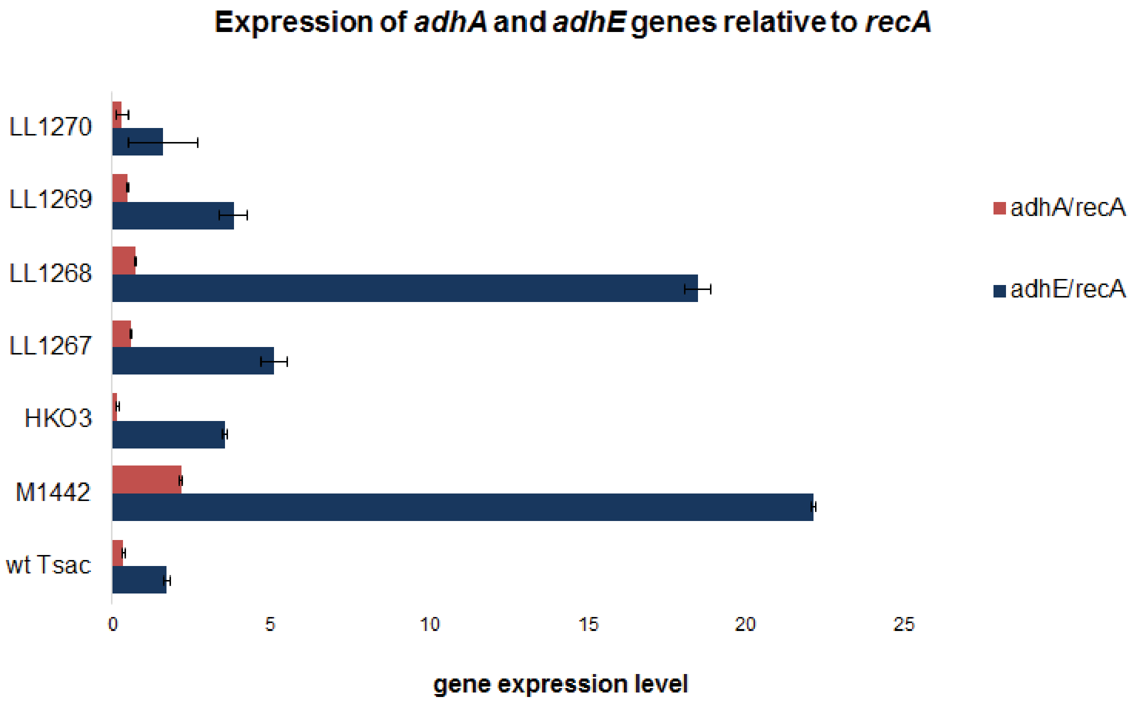

Results of RT-qPCR analysis of *adhA* and *adhE* genes in *T. saccharolyticum* strains normalized to *recA*. Error bars represents one standard deviation, n=2.
