# Supplementary figures and images for: Deletion of the hfsB gene increases ethanol production in Thermoanaerobacterium saccharolyticum and several other thermophilic anaerobic bacteria
Source: Biotechnol Biofuels. 2017 Nov 30;10:282. doi: 10.1186/s13068-017-0968-9 (PMC5707799; doi:10.1186/s13068-017-0968-9)

**Figure S1.** Confirmation of hydrogenase deletions in HKO strains

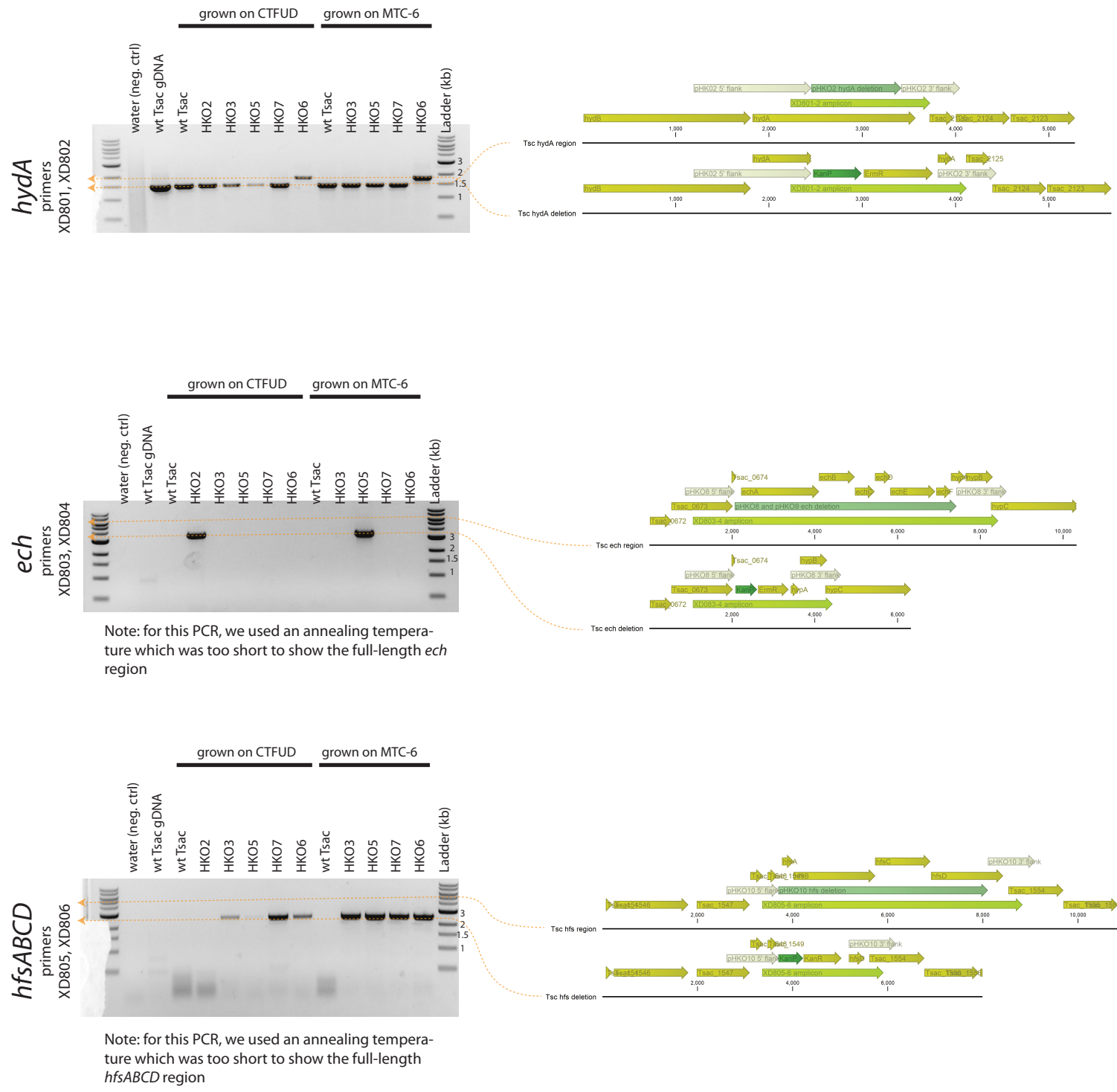

Supplement: Supplementary file 8 — Additional file 8: Figure S1. Confirmation of hydrogenase deletions in HKO strains. [file 13068_2017_968_MOESM8_ESM.pdf]
