# Supplementary material for: Deletion of the hfsB gene increases ethanol production in Thermoanaerobacterium saccharolyticum and several other thermophilic anaerobic bacteria
Source: Biotechnol Biofuels. 2017 Nov 30;10:282. doi: 10.1186/s13068-017-0968-9 (PMC5707799; doi:10.1186/s13068-017-0968-9)
Supplement: Supplementary file 9 — Additional file 9: Table S6. List of primers. [file 13068_2017_968_MOESM9_ESM.docx]

| ***T. saccharolyticum ∆hfsA::kan* Primers** |
| --- |
| hfsA Up Stream Flanking Fragment  AE2; 5’- CATGGCGTCGGGTCTTCCTGTC -3’  AE3; 5’- TGGGGTAAACATTTGTCAAGAAT CCGTATACTGTATAC -3’  *hfsA* KanR Fragment  AE1 ; 5’- ATTCTTGACAAATGTTTACCCCATTAG -3’  AE4 ; 5’- GCTTCTTTGAAATTAATGACACTCATATCCGATACAAATTCCTCGTAG -3’  *hfsA* Down Stream Flanking Fragment  AE5 ; 5’- ATGAGTGTCATTAATTTCAAAGAAGC -3’  AE6 ; 5’- GTA ATG CCG CTG AAA TCA CCG -3’  *hfsA* External Confirmation  AE24; 5’-TATTCTTTTCCACTTAAAGCTGTAG-3’  AE25; 5’- TGC CAA TCT TCG ACA ATA TCT C -3’ |
| ***T. saccharolyticum ∆hfsB::kan* Primers** |
| *hfsB* Up Stream Flanking Fragment  AE7; 5’- GGTCTTCCTGTCGTAGCAACAG-3’  AE8; 5’-GTAAACATTTGTCAAGAATTCATTTTAGTTCACC-3’ |
| *hfsB* KanR Fragment  AE1: kanR_F ; 5’- ATTCTTGACAAATGTTTACCCCATTAG -3’  AE9; 5’- GCAATATCGATGTAATGACTCATATCCGATACAAATTCCTC -3’ |
| *hfsB* Down Stream Flanking Fragment  AE10 ;5’- ATG AGT CAT TAC ATC GAT ATT GCA CAT G -3’  AE11; 5’- CACAATCCTGACCTTTATTCTAAGATCG -3’ |
| *hfsB* External Confirmation  AE26 ; 5’-ATGTTCTTAGGAAATTATCGAGAG-3’  AE27; 5’- CAT TGC GAC TTT AGT TCT CTG C -3’ |
| ***T. saccharolyticum ∆hfsC::kan* Primers** |
| *hfsC* Up Stream Flanking Fragment  AE12 ;5’- GAGAATTTAGAAGATGTTGACTCTG -3’  AE13 ;5’-GGTAAACATTTGTCAAGAATTCATTCATCATCACCTTG-3’ |
| *hfsC* KanR Fragment  AE1 ; 5’- ATTCTTGACAAATGTTTACCCCATTAG -3’  AE14;5’- ACAAGGCAATCTATCCTTTATCCGATACAAATTCCTCGTAG-3’ |
| *hfsC* Down Stream Flanking Fragment  AE15; 5’- AAAGGATAGATTGCCTTGTTTAAG-3’  AE16;5’- GCATCCAACACAGCAACCATCTC-3’ |
| *hfsC* External Confirmation  AE28; 5’-CTGTGAAGGCGCTATAGATGC-3’  AE29 ; 5’- TCC ACT TCC ACA TCT TGC TG -3’ |
| ***T. saccharolyticum ∆hfsD::kan* Primers** |
| *hfsD* Up Stream Flanking Fragment  AE17 ;5’- TGGATATGGGTTCTGAGCTAAGAG -3’  AE18;5’- CTAATGGGGTAAACATTTGTCAAGAATCTGAACATCTGTG-3’ |
| *hfsD* KanR Fragment  AE1 ; 5’- ATTCTTGACAAATGTTTACCCCATTAG -3’  AE19 ;5’- GAT GGA CCC ATT ATT TTT AAA ACT CAT CCG ATA CAA ATT CC -3’ |
| *hfsD* Down Stream Flanking Fragment  AE20; 5’-GAGTTTTAAAAATAATGGGTCCATC-3’  AE21; 5’-ATATTTGAGGAGCACCGTCGTC-3’ |
| *hfsD* External Confirmation  AE30; 5'- CTACTTTGACAACGCGAATAGTG -3'  AE31; 5’-GCAGCACCTCACCTCTTACAC-3’ |
| ***kan*R Gene Internal Primers** |
| AE22; 5’- GCA CTT TGA ACG GCA TGA TGG -3’  AE23; 5’- CCA TCA TGC CGT TCA AAG TGC -3’ |
| ***T. xylanolyticum ∆hfsB::kan* Primers** |
| *hfsB* Up Stream Flanking Fragment  AE51; 5’-TACGGACGAAACGGAAGGGAATTTGC-3’  AE52; 5’-GGGGTAAACATTTGTCAAGAATTTTAGTTCACCTAAAACATATTC-3’ |
| *hfsB* KanR Fragment  AE1; 5’-ATTCTTGACAAATGTTTACCCCATTAG-3’  AE53; 5’-GCAATATCGATGTAATGACATCCGATACAAATTCCTCGTAG-3’ |
| *hfsB* Down Stream Flanking Fragment  AE54; 5’-GATGTCATTACATCGATATTGC -3’  AE55; 5’-CCACTAAGAAATTTACGTCAGTAGCG-3’ |
| *hfsB* External Confirmation  AE56; 5’-GCAACAGACATTGGCGATTTAGG-3’  AE57; 5’-AGCTCTTCCACAATCCTGACC-3’ |
| ***T. thermosaccharolyticum ∆hfsB::kanR* Primers** |
| *hfsB* Up Stream Flanking Fragment  AE61 ;5’-GAGATGAGCATAATCTTTGAAAAACTGG-3’  AE62 ;5’-GGTAAACATTTGTCAAGAATTGTAAATTACCTAGCACATATTCTC-3’ |
| *hfsB* KanR Fragment  AE1; 5’-ATTCTTGACAAATGTTTACCCCATTAG-3’  AE58 ;5’-CAATATCAATGTAATGGCTCATATCCGATACAAATTCCTCG-3’ |
| *hfsB* Down Stream Flanking Fragment  AE63; 5’-ATGAGCCATTACATTGATATTGCAC-3’  AE64; 5’-GATCCGCAGGAAAATCAGGATTTTGATG-3’ |
| *hfsB* External Confirmation  AE73 ;5’-CGTGATAAAATGAGTATATACG-3’  AE74 ;5’-CTCTAATAAGTTAATAAGTTCTTCAAC-3’ |
| ***Thermoanaerobacter mathranii* subsp. mathranii str. A3 *∆hfsB::kanR* Primers** |
| *hfsB* Up Stream Flanking Fragment  AE65;5’-CACCTTACAGCATTCTATTGG-3’  AE66; 5’-GGTAAACATTTGTCAAGAATAATTATTCCTCCTCCCATTTAC-3’ |
| *hfsB* KanR Fragment  AE1; 5’-ATTCTTGACAAATGTTTACCCCATTAG-3’  AE59; 5’- CTTCAGCAAAGACCTTCATATCCGATACAAATTCCTC-3’ |
| *hfsB* Down Stream Flanking Fragment  AE67;5’-ATGAAGGTCTTTGCTGAAGTGTTCAC-3’  AE68; 5’-GTTGAGCGATTGACAAGCAGG-3’ |
| *hfsB* External Confirmation  AE75; 5’-GAGTGCCTCTATCCTATGTATGC-3’  AE76; 5’-GCCAGCTCTTCTACTACCTTGAG-3’ |
|  |
| ***T. saccharolyticum* ∆hfsAB::kanR primers** |
| hfsAB Up Stream Flanking Fragment  AE2; 5’- CATGGCGTCGGGTCTTCCTGTC -3’  AE3; 5’- TGGGGTAAACATTTGTCAAGAAT CCGTATACTGTATAC -3’ |
| *hfsAB* KanR Fragment:  AE1 ; 5’- ATTCTTGACAAATGTTTACCCCATTAG -3’  AE9; 5’- GCAATATCGATGTAATGACTCATATCCGATACAAATTCCTC -3’ |
| *hfsAB* Down Stream Flanking Fragment:  AE10 ;5’- ATG AGT CAT TAC ATC GAT ATT GCA CAT G -3’  AE11; 5’- CACAATCCTGACCTTTATTCTAAGATCG -3’ |
| *hfsAB* external confirmation  AE24; 5’-TATTCTTTTCCACTTAAAGCTGTAG-3’  AE27; 5’- CAT TGC GAC TTT AGT TCT CTG C -3’ |
| ***T. saccharolyticum* ∆hfsCD::kanR primers** |
| *hfsCD* Up Stream Flanking Fragment  AE12 ;5’- GAGAATTTAGAAGATGTTGACTCTG -3’  AE13 ;5’-GGTAAACATTTGTCAAGAATTCATTCATCATCACCTTG-3’ |
| *hfsCD* KanR Fragment:  AE1 ; 5’- ATTCTTGACAAATGTTTACCCCATTAG -3’  AE18;5’- CTAATGGGGTAAACATTTGTCAAGAATCTGAACATCTGTG-3’ |
| *hfsCD* Down Stream Flanking Fragment:  AE20; 5’-GAGTTTTAAAAATAATGGGTCCATC-3’  AE21; 5’-ATATTTGAGGAGCACCGTCGTC-3’ |
| *hfsCD* External Confirmation  AE28; 5’-CTGTGAAGGCGCTATAGATGC-3’  AE31; 5’-GCAGCACCTCACCTCTTACAC-3’ |
| ***C. thermocellum ∆hfsB* Primers** |
| 5’ Flanking Fragment |
| AE317; 5’-AGGCGTATCACGAGGCgatCCAGCAACTATTCAAAGGCC  AE318; 5’-TAGACTCGCGCTCCTTATC-3’ |
| 3’ Flanking Fragment |
| AE319; 5’-GATAAGGAGCGCGAGTCTAATGAATGATTTATGCGTTG-3’  AE320; 5’-CATGcCTATTCCCACgatCATTCTTGCTATTATCGAAG-3’ |
| Internal Region Fragment |
| AE323; 5’-CCTGGCCCAGTAGTTcagGCTTGGGTTTAAAGGTGCGG-3’  AE324; 5’-TTCACTACTATTAGcagAGACGGGCTTAAGTTC-3’ |
| Vector backbone I |
| AE321; 5’-atcGTGGGAATAGgCATG-3’  AE322; 5’-ctgAACTACTGGGCCAGG-3’ |
| Vector backbone II |
| AE315; 5’-ctgCTAATAGTAGTGAAAAAATC-3’  AE316; 5’-atcGCCTCGTGATACGCCT-3’ |
| 5’ flank-3’ falnk confirmation for pDGO145-hfsB in *E. coli* |
| AE115; 5’- CACCTGACGTCTAAGAAA-3’ |
| AE118: 5’- TCTTTTCCTCTCTTTCGG-3’ |
| Internal Region confirmation for pDGO145-hfsB in *E. coli* |
| AE119; 5’-TTTAAACCCGCTGATCCT-3’ |
| AE120; 5’-GTTGTCTAACTCCTTCCT-3’ |
| Marker Integrity |
| cat-hpt |
| AE198; 5’-GCTATCTTTACAGGTACATCATTCTGTTTGTG-3’ (616 bp)  AE199; 5’-TTTCATCAAAGTCCAATCCATAACCC-3’ |
| cbp-tdk |
| AE200; 5’-ACTTCATGGCACTTTCTACACCTTGC-3’  AE201;5’-TCGGAGTAAGGTGGATATTGATTTGC-3’ |
| Chromosomal Confirmation (External) |
| AE222: 5’-GTCTTGCCCATTTGCTTAAG-3’  AE223: 5’-CGC TTA TGA ATG AAA TCA GGC-3’ |
| Chromosomal Confirmation (Internal) |
| 224: 5'- CTG CTA ACA GGA CTT CCC -3'  225: 5’-GTGCTGCCATACGGTAAAC-3’ |
| ***T. saccharolyticum adhA* and *adhE* expression** |
| *T. saccharolyticum* recA qPCR primers  gblock:  GTT***GAAGCCTTAGTGCGAAGTGG***TGCTGTGGATGTGATCGTTATTGACTCTGTAGCTGCTCTCGTACCGAAAGCAGAGATAGATGGTGATATGGG***CGATGCACATGTTGGACTTC***AAG  recA qPCR F : 5’-GAAGCCTTAGTGCGAAGTGG-3’  recA qPCR R : 5’-GAAGTCCAACATGTGCATCG-3’  *T.saccharolyticum* adhA qPCR primers  AAT***AGCTCATGGTTTAGGGCTTG***GTGCAATATTGCCAGCAGTTATAAAAGCTATTTATCCAGCTACAGCAGAAGTATTGGCTGATGTATATAGTCCTATAGTTCCTGGTTTAAAAGGACTG***CCTGTTGAGGCGGAGTATGT***AGC  adhA qPCR F: 5’-AGCTCATGGTTTAGGGCTTG-3’  T.sacch. adhA qPCR R: 5’-ACATACTCCGCCTCAACAGG-3’  *T.saccharolyticum* adhE qPCR primers  TCA***GCTTCCATCCAAAGGCAATA***AAGTGCAGCATCGCAGCAGCCAAAGTGATGTATGAAGCTGCACTAAAGGCAGGCGCACCTGAAGGATGCATAGGATGGAT***AGAAACGCCATCAATTGAGG***CCA  adhE qPCR F: 5’-GCTTCCATCCAAAGGCAATA-3’  adhE qPCR R: 5’-CCTCAATTGATGGCGTTTCT-3’  Hydrogenase deletion confirmation primers  XD801: 5’-GTCGTCTCCTTCCATCGT-3’  XD802: 5’-CTTCATCTTCCCACTCCTTC-3’  XD803: 5’-GGCAAAAAGAGGCACAAAC-3’  XD804: 5’-CATCCTCATAACCCTCCAC-3’  XD805: 5’-GTGTTGTCGATTTGCTTG-3’  XD806: 5’-ATGTTCGATTTTGGTGGTT-3’ |
