# Supplementary material for: Deletion of the hfsB gene increases ethanol production in Thermoanaerobacterium saccharolyticum and several other thermophilic anaerobic bacteria
Source: Biotechnol Biofuels. 2017 Nov 30;10:282. doi: 10.1186/s13068-017-0968-9 (PMC5707799; doi:10.1186/s13068-017-0968-9)
Supplement: Supplementary file 10 — Additional file 10: Additional methods. Additional methods. [file 13068_2017_968_MOESM10_ESM.docx]

# Supplemental Methods

## Illumina sequencing

Whole genome resequencing was used to verify strain construction and check for secondary mutations. It was performed as previously described (1). Raw data is available from the JGI Sequence Read Archive (See accession numbers in Table 1). With DNA as the input, whole genome sequencing was used to identify mutations. With RNA as the input, whole genome sequencing (RNAseq) was used to determine gene expression. Briefly, RNA or DNA was submitted to the Joint Genome Institute (JGI) for sequencing with an Illumina MiSeq instrument. When RNA was submitted it was first converted to cDNA as follows: rRNA was removed from 100 ng of total RNA using Ribo-Zero(TM) rRNA Removal Kit (Epicentre). Stranded cDNA libraries were generated using the Illumina Truseq Stranded RNA LT kit. The rRNA depleted RNA was fragmented and reversed transcribed using random hexamers and SSII (Invitrogen) followed by second strand synthesis. The fragmented cDNA was treated with end-pair, A-tailing, adapter ligation, and 10 cycles of PCR. The prepared library was quantified using KAPA Biosystem’s next-generation sequencing library qPCR kit and run on a Roche LightCycler 480 real-time PCR instrument. The quantified library was then pool with other libraries and prepared for sequencing on the Illumina HiSeq 2500 sequencing platform utilizing a TruSeq Rapid paired-end cluster kit, v2, with the HiSeq2500 sequencer instrument to generate a clustered flow-cell for sequencing. Sequencing of the flow-cell was performed on the Illumina HiSeq2500 sequencer using HiSeq Rapid SBS sequencing kits, v2, following a 2x150 indexed run recipe.

Unamplified libraries were generated using a modified version of Illumina’s standard protocol. 100 ng of DNA was sheared to 500 bp using a focused ultrasonicator (Covaris). The sheared DNA fragments were size-selected using SPRI beads (Beckman Coulter). The selected fragments were then end-repaired, A-tailed, and ligated to Illumina compatible adapters (IDT, Inc.) using KAPA- Illumina library creation kit (KAPA biosystems). Libraries were quantified using KAPA Biosystems’ next-generation sequencing library qPCR kit and run on a Roche LightCycler 480 real-time PCR instrument. The quantified libraries were then multiplexed into pools for sequencing. The pools were loaded and sequenced on the Illumina MiSeq sequencing platform utilizing a MiSeq Reagent Kit v2 (300 cycle) following a 2 × 150 indexed run recipe. Paired-end reads were generated, with an average read length of 150 bp and paired distance of 500 bp. Data was analyzed with CLC Genomics Workbench v10 (Qiagen).

## RT-qPCR analysis

To determine gene expression by RT-qPCR analysis, cDNA was synthesized from 500 ng of RNA using the iScript cDNA synthesis kit (Bio-Rad, Hercules, CA, USA). Expressions levels were measured by CFX96 qPCR system (Bio-Rad, Hercules, CA, USA) with SsoFast™ EvaGreen® Supermix (Bio-Rad). 55 °C used as annealing temperature to determine expression levels of adhE and adhA genes in *T. saccharolyticum* strains. In each case, expression was normalized to *recA* RNA levels. Removal of contaminating DNA from RNA samples confirmed via cDNA synthesis in the presence and absence of reverse transcriptase followed by qPCR using *recA* primers to ensure only background levels were detected in the samples lacking reverse transcriptase. Standard curves were generated using a synthetic DNA template (gBlock, IDT, Coralville, IA, USA) containing the amplicons. Primers used for qPCR are listed in Table S6

## Proteomic analysis

Wild-type and Δ*hfs* strains were processed for LC-MS/MS analysis as previously described (2). Briefly, cell pellets from 50 ml of culture were re-suspended in SDS lysis buffer (4% SDS, 100 mM Tris-HCl, pH 8.0), boiled, sonicated, and crude protein lysate quantified via BCA assay. Two milligrams of crude protein were adjusted to 25 mM dithiothreitol (DTT), boiled, then precipitated with trichloroacetic acid (TCA). The protein pellet was washed with cold acetone then air dried before resuspension in 8 M urea, 100 mM Tris-HCl, 5 mM DTT, pH 8.0. Cysteines were then alkylated with 15 mM iodoacetamide (IAA) before digestion with sequencing-grade porcine trypsin (1:100 [w/w] overnight at room temperature after dilution to 4 M urea followed by another 1:100 [w/w] addition for 4 hr after dilution to 2 M urea). Digests were then salted and acidified and tryptic peptides collected via filtration through a 10 kDa MWCO spin column (Vivaspin 2; GE Healthcare).

Five micrograms of tryptic peptides were loaded onto a biphasic (reversed-phase [RP] and strong-cation exchange [SCX]) MudPIT back column, washed, and placed in-line with an in-house pulled nanospray emitter packed with 15 cm of RP resin as previously described (3). Peptides were separated by a 2-step mini-MudPIT LC-MS/MS analysis employing 50 mM and 500 mM ammonium acetate salt cuts followed by standard, two-hour RP gradients. Eluting peptides were measured and sequenced in real time by a hybrid LTQ-XL Orbitrap mass spectrometer operating in data-dependent acquisition (DDA) mode. Relevant DDA parameters include: 1 full scan (15k resolution) followed by 10 MS/MS scans; isolation width = 2.2 m/z; CID energy = 35%; dynamic exclusion residence time and window = 30 s and 20 ppm, respectively; monoisotopic precursor selection = on; charge state screening = reject unassigned charges. Resulting MS/MS spectra were searched against the *Thermoanaerobacterium saccharolyticum* proteome database, concatenated with common contaminants and reversed sequences to assess false-discovery rates, with the Myrimatch v. 2.1 search algorithm (4). Peptide-spectral matches (PSM) were scored, filtered (peptide-level FDR < 1 %), and assigned to proteins via IDPicker v. 3.0 (5). Identified proteins were quantified by MS1 intensity via IDPicker’s label-free quantitation method and normalized across runs to evaluate proteins that had marked differences in abundance due to specific deletions in genes of the *Hfs* operon.

## Supplemental References

1. Zhou J, et al. (2015) Physiological roles of pyruvate ferredoxin oxidoreductase and pyruvate formate-lyase in *Thermoanaerobacterium saccharolyticum* JW/SL-YS485. *Biotechnol Biofuels* 8(1):138.

2. Tian L, et al. (2016) Simultaneous achievement of high ethanol yield and titer in *Clostridium thermocellum*. *Biotechnol Biofuels* 9(1):116.

3. Giannone RJ, et al. (2014) Life on the edge: functional genomic response of Ignicoccus hospitalis to the presence of *Nanoarchaeum equitans*. *ISME J* 9(1):101–14.

4. Tabb DL, Fernando CG, Chambers MC (2008) MyriMatch:highly accurate tandem mass spectral peptide identificaiton by multivariate hypergeometric analysis. *J Proteome Res* 6(2):654–661.

5. Ma ZQ, et al. (2009) IDPicker 2.0: Improved protein assembly with high discrimination peptide identification filtering. *J Proteome Res* 8(8):3872–3881.
